# Supplementary material for: Structure of the planar cell polarity cadherins Fat4 and Dachsous1
Source: Nat Commun. 2023 Feb 16;14:891. doi: 10.1038/s41467-023-36435-x (PMC9935876; doi:10.1038/s41467-023-36435-x)
Supplement: Supplementary file 1 — Supplementary Information [file 41467_2023_36435_MOESM1_ESM.pdf]

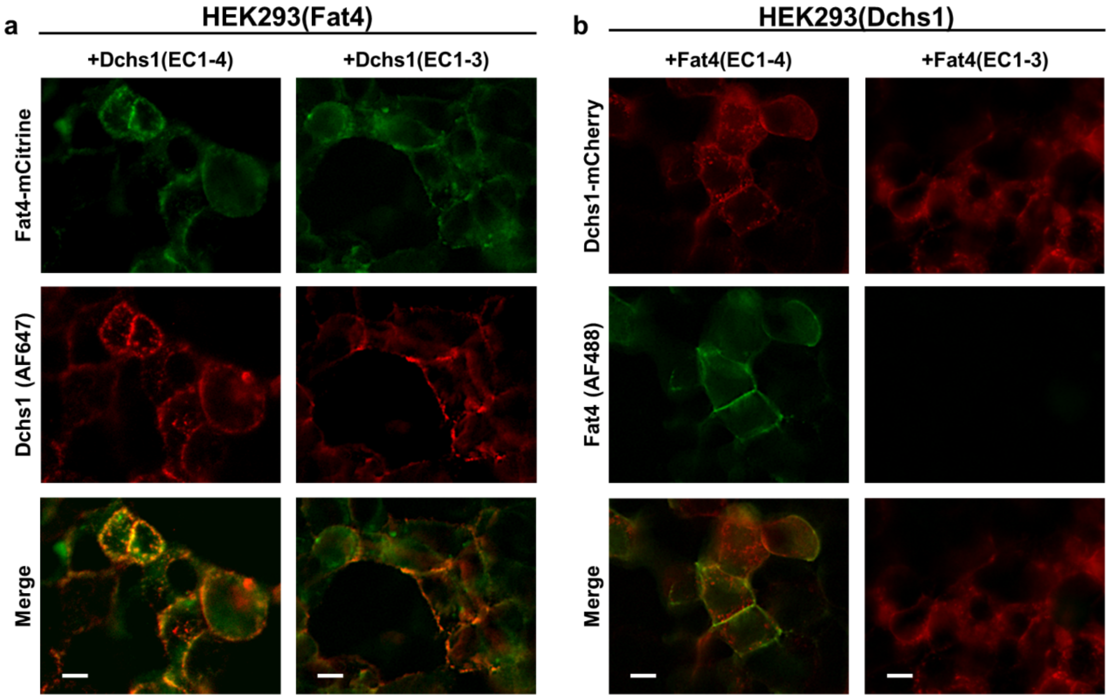

Supplementary Fig. 1: Cellular binding of Fat4 and Dchs1 truncation constructs.

HEK293 cells stably transfected with either Fat4-mCitrine or Dchs1-mCherry were stained using fluorescently labeled tetramers of various N-terminal truncations, fixed, and imaged to observe binding. **a.** Fat4-mCitrine cells stained with Dchs1(EC1-4) or Dchs1(EC1-3) tetramers. **b.** Dchs1-mCherry cells stained with Fat4(EC1-4) or Fat4(EC1-3) tetramers. Note the absence of staining using Fat4(EC1-3) tetramers. Each staining experiment was performed twice. Scale bar = 10 um.

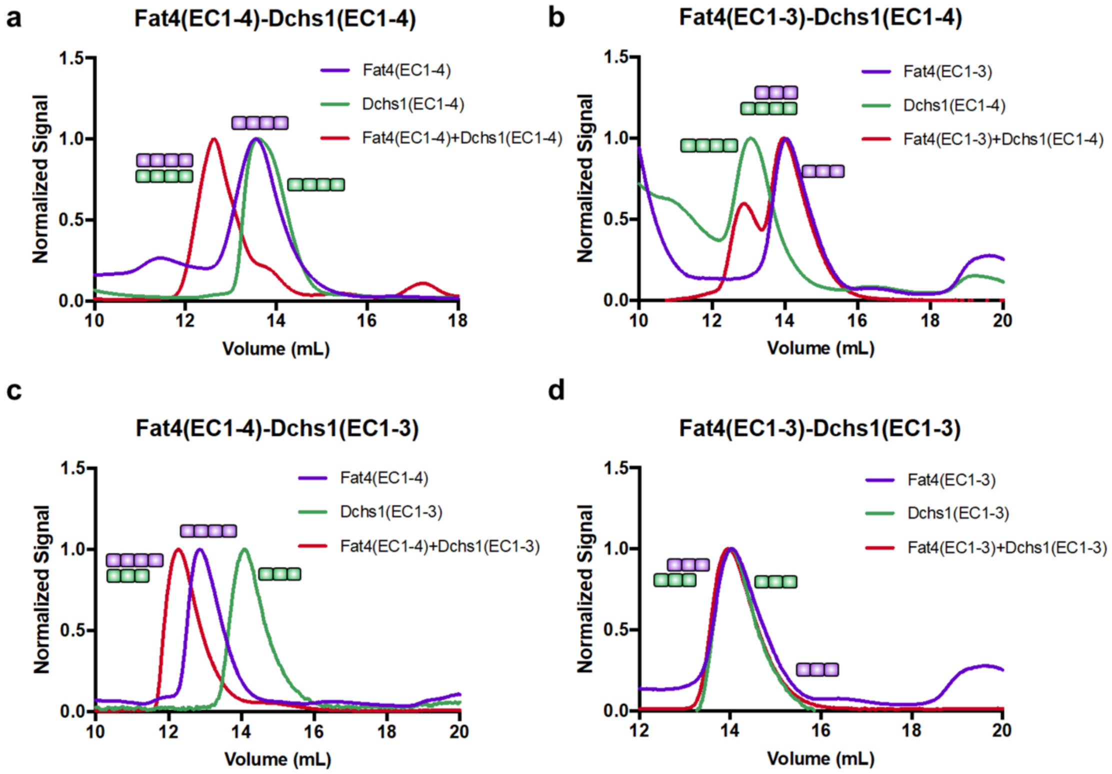

Supplementary Fig. 2: Purification of Fat4 and Dchs1 constructs.

Gel filtration chromatograms of various truncated Fat4 or Dchs1 constructs, either alone or in complex. For all complexes, proteins were pre-mixed at a 1:1 molar ratio prior to injection on the column. **a.** Purification of: Fat4(EC1-4), Dchs1(EC1-4), and Fat4(EC1-4)-Dchs1(EC1-4) complex. **b.** Purification of Fat4(EC1-3), Dchs1(EC1-4), and Fat4(EC1-3)-Dchs1(EC1-4) complex. **c.** Purification of Fat4(EC1-4), Dchs1(EC1-3), and Fat4(EC1-4)-Dchs1(EC1-3) complex. **d.** Purification of Fat4(EC1-3), Dchs1(EC1-3), and Fat4(EC1-4)-Dchs1(EC1-3). Note that co-elution was not observed for Fat4(EC1-3)-Dchs1(EC1-4) or Fat4(EC1-3)-Dchs1(EC1-3).

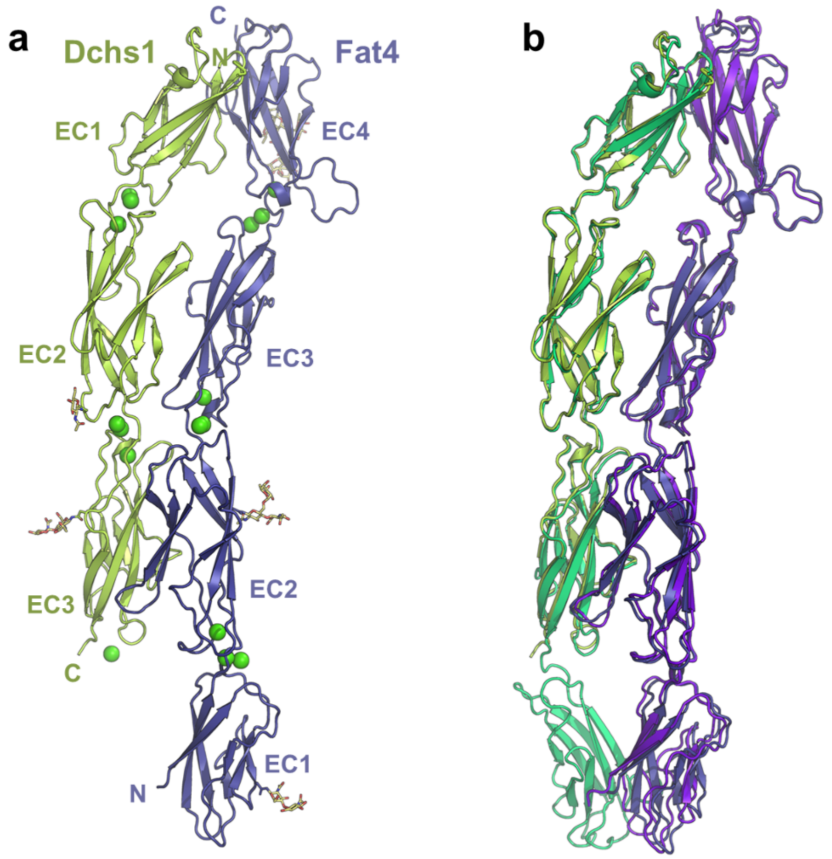

Supplementary Fig. 3: Comparison of Fat4:Dchs1 high- and low-resolution structures.

a. High-resolution crystal structure of Fat4(EC1-4) (in blue) in complex with Dchs1(EC1-3) (in yellow-green) in cartoon representation. N-linked glycans are colored yellow and Ca^{2+} ions are colored green. **b.** Global alignment of low- and high-resolution structures exhibits limited interdomain flexibility.

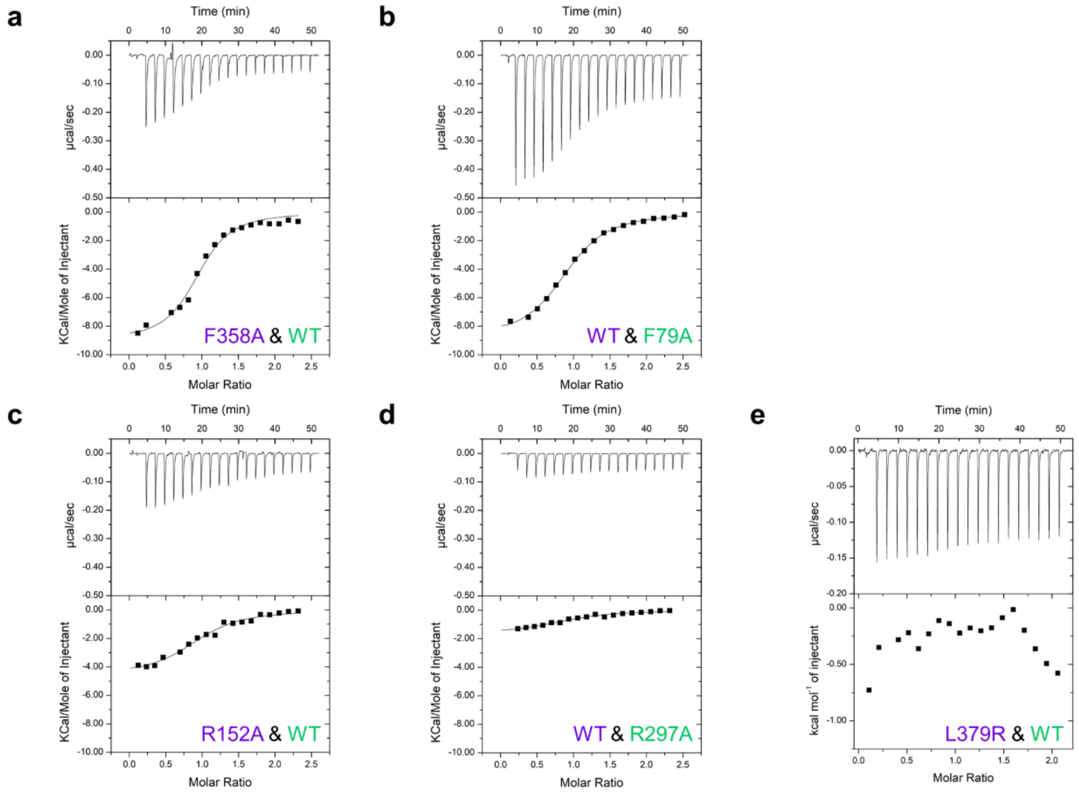

Supplementary Fig. 4: Binding of Fat4 and Dchs1 interface mutants.

ITC was used to measure the binding of various Fat4 and Dchs1 interface mutants. All mutants were generated in Fat4(EC1-4) or Dchs1(EC1-4) constructs. **a.** ITC binding isotherm for Fat4^{F358A} and Dchs1. **b.** ITC binding isotherm for Fat4 and Dchs1^{F79A}. **c.** ITC binding isotherm for Fat4^{R152A} and Dchs1. **d.** ITC binding isotherm for Fat4 and Dchs1^{R297A}. **e.** ITC binding isotherm for Fat4^{L379R} and Dchs1.

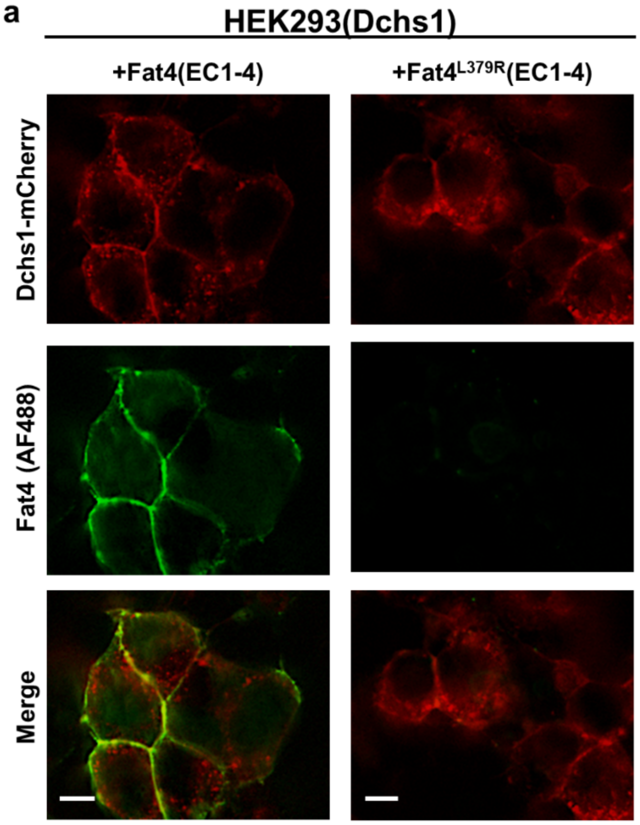

Supplementary Fig. 5: Staining of Dchs1-expressing cells with Fat4^{L379R} mutant.

a. Dchs1-expressing cells were incubated with fluorescently labeled tetramers of Fat4(EC1-4) or Fat4^{L379R}(EC1-4), fixed and imaged. Fat4 tetramers, but not Fat4L379R tetramers, bound to the cells. Each staining experiment was performed twice. Scale bar = 10 um.

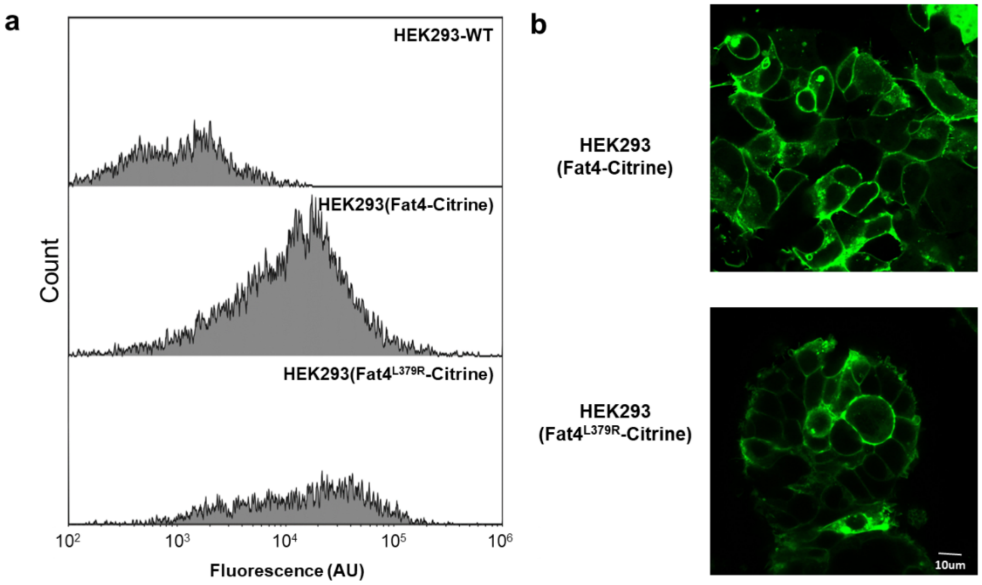

Supplementary Fig. 6: FACS analysis of fluorescently tagged proteins.

a. Comparison of the fluorescence of HEK293 cells expressing either Fat4 or Fat4^{L379R} tagged with Citrine (as indicated for each row). Control cells (HEK293-WT) that do not express fluorescent protein are shown in the top row. **b.** Confocal imaging of HEK293 cells expressing either Fat4 or Fat4^{L379R} tagged with Citrine. There is no difference in surface localization between the two constructs. Flow cytometry and imaging was performed twice.

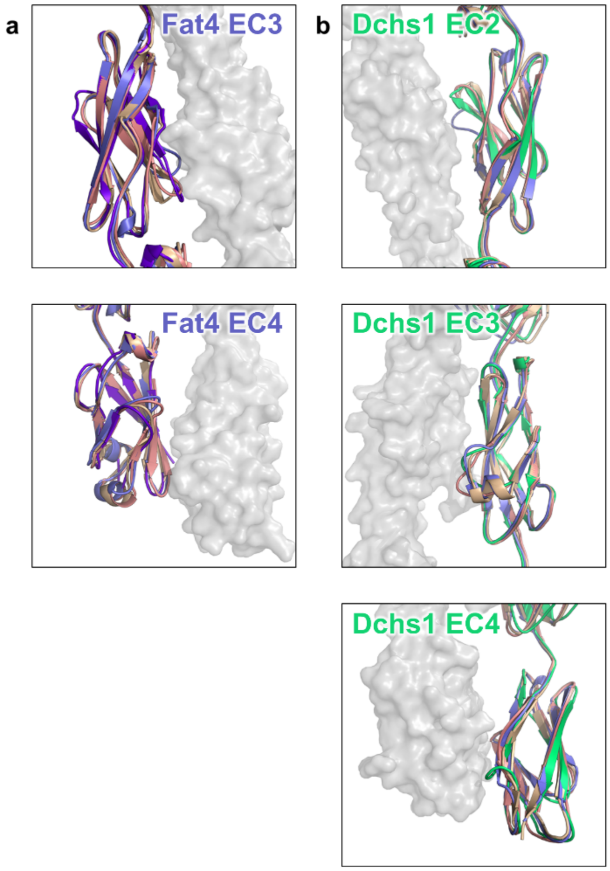

Supplementary Fig. 7: Alignment of Fat4, Dchs1, and protocadherin EC domains.

a,b. Fat4 and Dchs1 EC domains were superimposed with analogous domains from protocadherins α4 (in blue), β6 (in salmon), and γB3 (in light yellow). For Fat4 alignments, Dchs1 is depicted as a grey surface representation. For Dchs1 alignments, Fat4 is depicted as a grey surface representation.

a

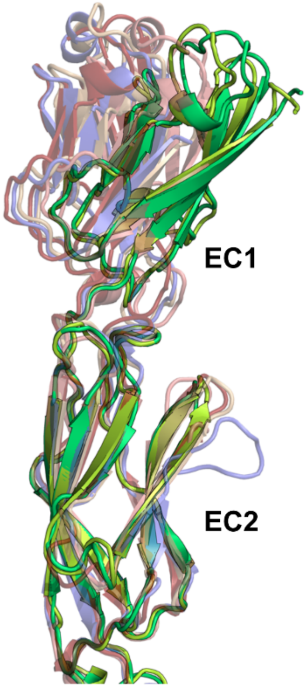

b

Protocadherins	β5-β6 Loop Region
α4	I L F V N S R I D R E E L
β6	N L L L K E K P D R E A L
γB3	N L L V S D R I D R E E I
17	Y T K - - Q R I D R E S L
Fat4	A L Y T T S T I D R E S L
Dchs1	V V R T A R V L D R E Q R

β5 Ca^{2+}

Protocadherins	β5-β6 Loop Region
α4	C G R S A E C S I H L
β6	C G A T E P C - V L H
γB3	C G K K S T C - V L E
17	C R H N A K C - Q L S
Fat4	P S D V - - - - I N L
Dchs1	D R - - - - - - Y R F

β6

Supplementary Fig. 8: Dchs1(EC1) exhibits a greater degree of freedom.

a. Aligning EC2 of protocadherins α4 (in blue), β6 (in salmon), γB3 (in light yellow), 17 (in red), and Dchs1 from the low- (in green) and high-resolution (in yellow-green) structures shows the characteristic tilt is unique to Dchs1. **b.** Structural alignment of the β5-β6 loop regions for the protocadherins compared in this paper. The conserved D-R-E motif that coordinates calcium is highlighted in green. Protocadherin subfamilies all contain a disulfide-linked loop that is absent in Fat4 and Dchs1.

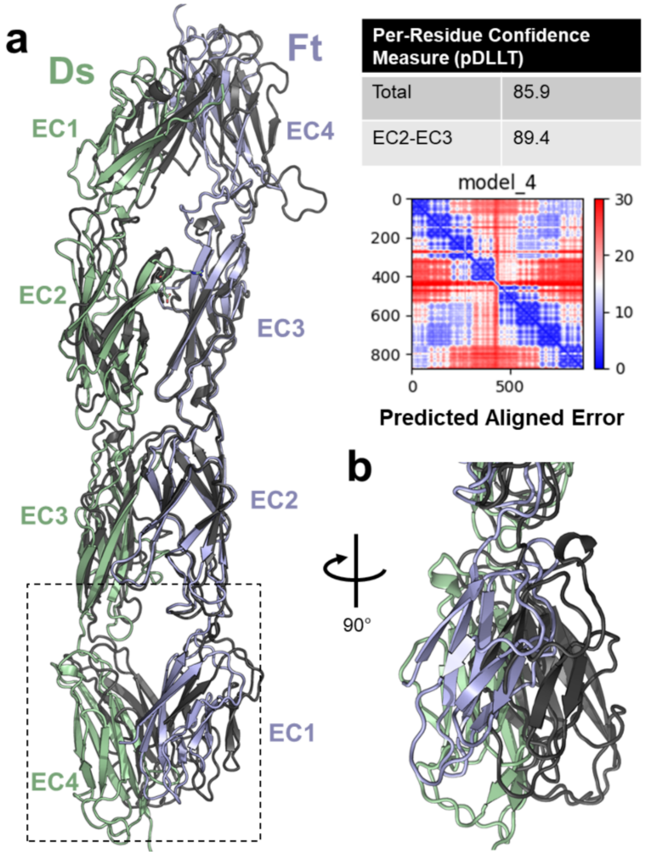

Supplementary Fig. 9: *Drosophila* Ft(EC1-4):Ds(EC1-4) Alphafold model is structurally similar to Fat4(EC1-4):Dchs1(EC1-4).

a. Out of five structure predictions, the top three models did not predict meaningful interactions between Ft and Ds. The fourth and fifth ranking predictions were structurally similar to the human Fat4:Dchs1 complex (shown in black). The per-residue confidence measure in the EC2-EC3 region, which contains Fj phosphorylation sites, was higher than that of the overall structure. **b.** The most substantial difference between the Ft:Ds model and the Fat4:Dchs1 structure (black) was the skewed position of the Ft(EC1) and Ds(EC4) domains.

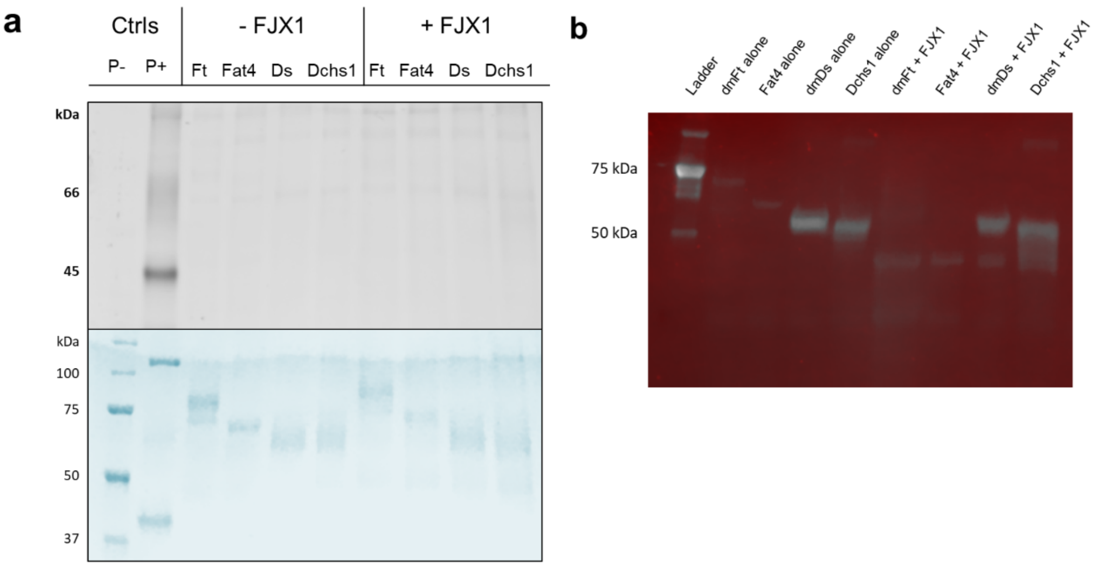

Supplementary Fig. 10: Fat4 and Dchs1 proteins were not phosphorylated when expressed in 293-ES cells.

a. His-tagged dmFt(EC1-4), Fat4(EC1-4), dmDs(EC1-4), or Dchs1(EC1-4) proteins were expressed in 293-ES cells using the BacMam system. All proteins were either expressed alone or co-expressed with full-length FJX1 (also containing a 6xHis-tag). Proteins were analyzed by SDS-PAGE (bottom) and a phosphostain was used to detect phosphorylation (top). No proteins were phosphorylated under any conditions tested. Unphosphorylated and phosphorylated protein ladders were used as controls. **b.** For conditions with FJX1 co-expression, the presence of FJX1 was detected by Western blotting with an antibody against the 6x-His tag. Experiments were performed twice. Uncropped images can be found in **Supplementary Figure 11**.

Supplementary Fig. 10a

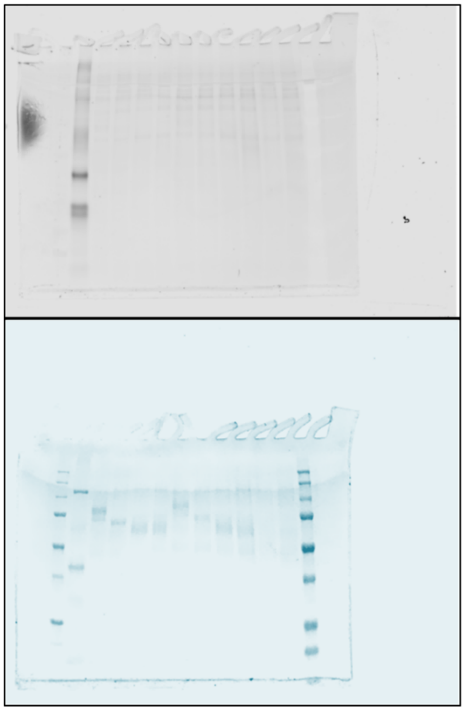

Supplementary Fig. 10b

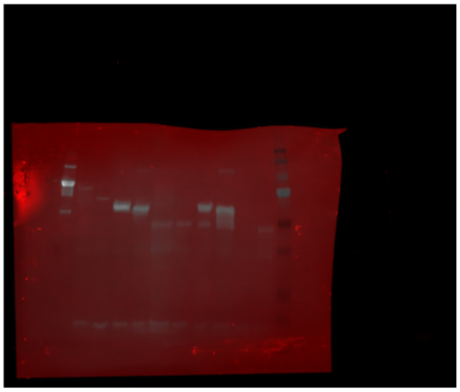

Supplementary Figure 11: Uncropped gels and blots from this Supplementary file.

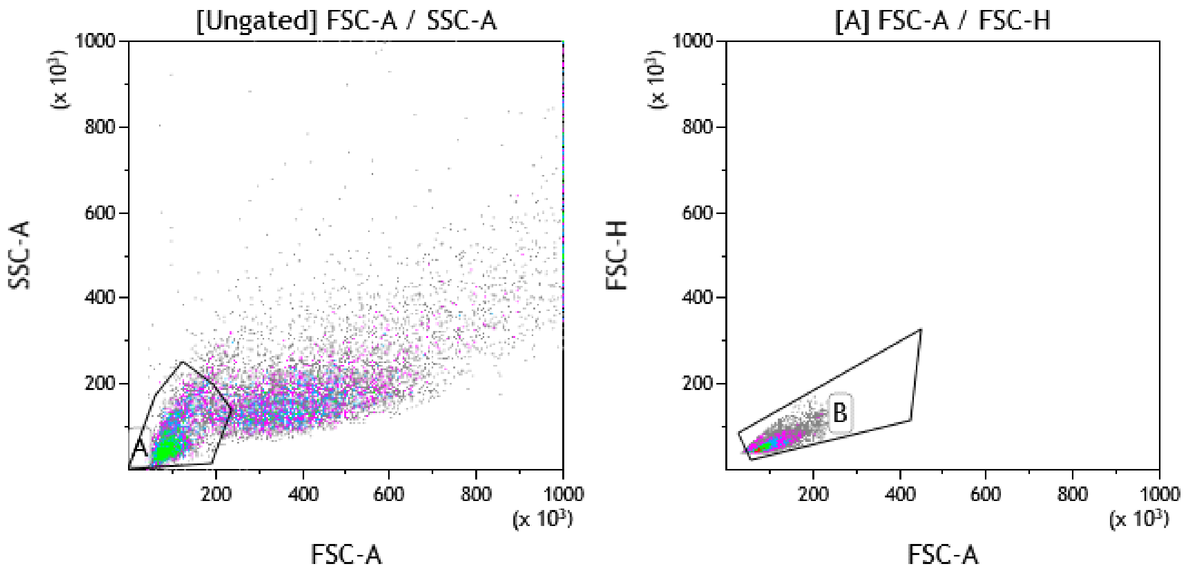

Supplementary Figure 12: Fluorescence-activated cell sorting (FACS) Gating Strategy for Supplementary Figure 6.

Cells are separated from debris by a forward versus side scatter defined in area marked as A **(left)**. Single cells are identified by plotting forward scatter-area against forward scatter-height in area marked as B **(right)**.

	Fat4(EC1-4)-Dchs1(EC1-3)	Fat4(EC1-4)-Dchs1(EC1-4)
**Resolution range (Å)**	48.29 - 2.3 (2.382 - 2.3)	41.92 - 3.688 (3.82 - 3.688)
**Space group**	P 1 21 1	P 21 21 21
**Unit cell**<br>**a, b, c (Å)**<br>**α, β, γ (°)**	 88.658 61.372 100.973<br>90 112.736 90	 64.222 82.102 224.759<br>90 90 90
**Total reflections**	154058 (15510)	23181 (1746)
**Unique reflections**	44794 (4416)	12968 (1214)
**Multiplicity**	3.4 (3.5)	2.0 (2.0)
**Completeness (%)**	99.88 (100.00)	95.90 (91.83)
**Mean I/sigma(I)**	10.26 (1.38)	4.05 (2.33)
**Wilson B-factor**	48.97	73.78
R_{merge}	0.1313 (0.9528)	0.03454 (0.01637)
R_{meas}	0.1551 (1.125)	0.04885 (0.02314)
R_{pim}	0.08192 (0.5942)	0.03454 (0.01637)
**CC1/2**	0.986 (0.582)	0.994 (0.998)
**Reflections used in refinement**	44762 (4417)	12896 (1203)
**Reflections used for R-free**	2008 (198)	620 (58)
R_{work}	0.1901 (0.3016)	0.2709 (0.3003)
R_{free}	0.2357 (0.3460)	0.2978 (0.3107)
**Number of non-hydrogen atoms**	6178	6485
**Macromolecules**	5626	6383
**Ligands**	260	102
**Solvent**	292	
**Protein residues**	741	852
**RMS(bonds)**	0.009	0.003
**RMS(angles)**	1.31	0.68
**Ramachandran favored (%)**	96.61	92.04
**Ramachandran allowed (%)**	2.85	7.13
**Ramachandran outliers (%)**	0.54	0.83
**Rotamer outliers (%)**	0.00	0.00
**Clashscore**	5.79	10.86
**Average B-factor ($Å^2$)**	63.57	72.45
**macromolecules**	61.52	72.26
**ligands**	106.03	84.83
**solvent**	65.38	
**Number of TLS groups**	7	

Supplementary Table 1: Data collection and refinement statistics. Statistics for the highest-resolution shell are shown in parentheses.

	Chain	Z-score	RMSD	Aligned	Residues	Sequence Identity (%)	PDB Description
**1**	5k8r-A	27.2	3	397	414	34	PROTOCADHERIN GAMMA-B3;
**2**	6mer-A	26.9	3.2	397	414	34	PROTOCADHERIN GAMMA-B3;
**3**	6vg1-B	26.8	6.5	404	641	36	PROTOCADHERIN PROTEIN;
**4**	6vft-B	26.8	5.5	397	414	35	PROTOCADHERIN-17;
**5**	6bx7-A	26.7	3.4	401	429	32	PROTOCADHERIN-1;
**6**	6vft-A	26.6	5.5	398	413	34	PROTOCADHERIN-17;
**7**	6meq-A	26.6	3.3	398	416	33	PROTOCADHERIN GAMMA-B3;
**8**	6vfp-A	26.5	3.4	404	439	32	PROTOCADHERIN-1;
**9**	6vft-C	26.3	5.8	403	420	33	PROTOCADHERIN-17;
**10**	6mga-A	26.2	3.3	398	429	32	PROTOCADHERIN-1;
**11**	6vfr-B	26.2	4.9	400	428	30	PROTOCADHERIN-18;
**12**	6vfu-B	26.1	4.1	393	411	36	PROTOCADHERIN-19;
**13**	6vfu-C	26	4.7	400	428	35	PROTOCADHERIN-19;
**14**	6vfr-A	26	4.6	400	428	30	PROTOCADHERIN-18;
**15**	6vfu-A	25.7	4.9	401	428	35	PROTOCADHERIN-19;
**16**	5szm-A	25.7	6.2	396	416	34	PCDHGA8
**17**	6vfw-A	25.5	5.5	397	423	33	PROTOCADHERIN-10;
**18**	6vfw-E	25.5	5	397	422	33	PROTOCADHERIN-10;
**19**	5dzy-A	25.5	4.3	393	414	32	PCDHB8 PROTEIN;
**20**	5dzw-A	25.5	5.6	391	417	35	PROTOCADHERIN ALPHA-4;
**21**	6vfw-D	25.4	5.2	396	423	33	PROTOCADHERIN-10;
**22**	6vfw-B	25.3	5.3	397	423	33	PROTOCADHERIN-10;
**23**	5szp-A	25.3	5.4	391	409	35	PROTOCADHERIN GAMMA B7;
**24**	5dzy-B	25.3	5.2	396	415	32	PCDHB8 PROTEIN;
**25**	5dzy-D	25.3	5.5	392	412	32	PCDHB8 PROTEIN;
**26**	6vfw-C	25.2	5.2	398	429	33	PROTOCADHERIN-10;
**27**	5iu9-A	25.2	4.5	398	417	32	PROTOCADHERIN-19 ISOFORM 1;
**28**	5szl-C	25.2	5.4	395	418	34	PROTOCADHERIN GAMMA A1
**29**	5szl-B	25.1	6	392	412	32	PROTOCADHERIN GAMMA A1
**30**	5dzx-A	25.1	5.5	392	414	32	PROTOCADHERIN BETA 6;

Supplementary Table 2: DALI server results for structures similar to Fat4(EC1-4).

	Chain	Z-score	RMSD	Aligned	Residues	Sequence Identity (%)	PDB Description
**1**	6vft-D	24.6	3.6	304	418	36	PROTOCADHERIN-17;
**2**	6vfw-D	23.9	3.7	300	423	32	PROTOCADHERIN-10;
**3**	6vg4-A	23.9	3.9	303	646	32	PROTOCADHERIN-10;
**4**	6vfq-A	23.8	3.4	300	424	32	PROTOCADHERIN-10;
**5**	4zpm-B	23.7	5.4	298	314	35	PROTEIN PCDHAC2;
**6**	4zpq-A	23.6	4.6	301	315	32	MCG133388, ISOFORM
**7**	4zi9-B	23.6	5.7	298	309	34	MCG133388, ISOFORM
**8**	4zpq-B	23.5	4.4	298	312	31	MCG133388, ISOFORM
**9**	4zi9-A	23.5	5.6	296	309	34	MCG133388, ISOFORM
**10**	5iu9-B	23.4	2.9	303	422	31	PROTOCADHERIN-19 I
**11**	4zpn-A	23.3	5.1	300	320	31	MCG133388, ISOFORM
**12**	6vfr-A	23.3	3.7	305	428	32	PROTOCADHERIN-18;
**13**	4zpn-B	23.3	4.5	300	320	32	MCG133388, ISOFORM
**14**	4zi8-A	23.2	3.9	299	313	36	PROTEIN PCDHGC3;
**15**	4zi8-B	23.2	4.2	306	323	36	PROTEIN PCDHGC3;
**16**	4zpp-A	23.2	4.7	297	311	31	MCG133388, ISOFORM
**17**	4zpo-A	23.2	5.2	297	311	31	MCG133388, ISOFORM
**18**	6vft-C	23.2	3.9	302	420	36	PROTOCADHERIN-17;
**19**	6vft-B	23.2	4	302	414	36	PROTOCADHERIN-17;
**20**	4zpl-A	23.1	4	300	316	34	PROTEIN PCDHB1;
**21**	6vfu-C	23.1	2.3	305	428	35	PROTOCADHERIN-19;
**22**	6vfu-A	22.9	2.1	304	428	35	PROTOCADHERIN-19;
**23**	6vft-A	22.9	3.9	285	413	38	PROTOCADHERIN-17;
**24**	4zpp-B	22.8	4.9	297	311	32	MCG133388, ISOFORM
**25**	6vfu-B	22.8	2.1	297	411	36	PROTOCADHERIN-19;
**26**	6vfr-B	22.7	3.3	306	428	32	PROTOCADHERIN-18;
**27**	5dzx-B	22.7	3.7	296	411	32	PROTOCADHERIN BETA
**28**	6vfw-C	22.7	3.8	301	429	32	PROTOCADHERIN-10;
**29**	6vfw-E	22.6	3.3	300	422	32	PROTOCADHERIN-10;
**30**	5szp-B	22.5	3.7	294	409	34	PROTOCADHERIN GAMM

Supplementary Table 3: DALI server results for structures similar to Dchs1(EC1-4).

Cadherin	EC1/EC4 Buried Surface Area ($Å^2$)	Shape Complementarity (S_c)	Hydrogen Bonds	Salt Bridges
Fat4/Dachsous1	1166 (54%)	0.582	18	14
γB3	344 (22%)	0.619	10	6
Pcdh-17(A:B)	346 (23%)	0.445	4	7
α4	1077 (50%)	0.571	14	8
β6	974 (43%)	0.508	22	3
E-Cadherin	-	0.839	10	4
Protocadherin 15/Cadherin 23	-	0.512	3	0

Supplementary Table 4: Interface analysis of representative cadherin superfamily structures.

Species used for ConSurf Analysis		
Homo sapiens (Human)	*Sus scrofa* (Wild Boar)	*Physeter catodon* (Sperm Whale)
Mus musculus (Mouse)	*Ailuropoda melanoleuca* (Giant Panda)	*Orcinus orca* (Orca Whale)
Xenopus laevis (African Clawed Frog)	*Ovis aries* (Sheep)	*Odobenus rosmarus* (Walrus)
Danio rerio (Zebrafish)	*Gallus gallus* (Red Junglefowl)	*Delphinapterus leucas* (Beluga Whale)
Drosophila melanogaster (Common Fruit Fly)	*Bos Taurus* (Domesticated Cow)	*Callorhinus ursinus* (Northern Fur Seal)
Myotis lucifugus (Little Brown Bat)	*Equus caballus* (Horse)	*Astyanax mexicanus* (Blind Cave Fish)

Supplementary Table 5: List of species used for ConSurf analysis. Species names are in italics.

ITC Experiment	Chi^2/DOF	N (sites)	deltaH (cal/mol)	deltaS (cal/mol K)	deltaG (cal/mol)
Fat4(EC1-4)-Dchs1(EC1-4) (**Figure 1C**)	14120	1.06 ± 0.0114	-3912 ± 62.21	15.5	-8533
Fat4(EC1-4)-Dchs1(EC1-3) (**Figure 1C**)	34430	0.978 ± 0.0137	-6219 ± 118	6.64	-8199
$Fat4^{F358A}$(EC1-4)-Dchs1(EC1-4) (**Extended Figure 3A**)	34950	0.915 ± 0.0119	-8124 ± 156.3	0.296	-8212
Fat4(EC1-4)-$Dchs1^{F79A}$(EC1-4) (**Extended Figure 3B**)	9113	0.924 ± 0.00978	-8843 ± 132.2	-4.01	-7647
$Fat4^{R152A}$(EC1-4)-Dchs1(EC1-4) (**Extended Figure 3C**)	32880	0.965 ± 0.0359	-4600 ± 236.5	9.6	-7462
Fat4(EC1-4)-$Dchs1^{R297A}$(EC1-4) (**Extended Figure 3D**)	3920	1.02 ± 0.138	-2910 ± 583.7	11.9	-6458
PhosFt(EC1-4)-Ds(EC1-4) (**Figure 6B**)	38780	0.777 ± 0.0242	5097 ± 266.3	42.1	-7455
PhosFt(EC1-4)-PhosDs(EC1-4) (**Figure 6B**)	27550	0.751 ± 0.0514	7268 ± 749.7	46.6	-6626

Supplementary Table 6: Fitting parameters for ITC experiments displayed within this paper

Fat4F358A Forward Primer	cggtagtgaagttccgctac**GC**cccggccacctcgcgctacg
Fat4F358A Reverse Primer	cgtagcgcgaggtggccggg**GC**gtagcggaacttcactaccg
Fat4L379R Forward Primer	aagtgggcaccgtggtggctc**G**gctcaccgtgacggacgcaga
Fat4L379R Reverse Primer	tctgcgtccgtcacggtgagc**C**gagccaccacggtgcccactt
Fat4R152A Forward Primer	ggaagacagtagcagcgga**GC**ccaagtcatcttagacaccg
Fat4R152A Reverse Primer	cggtgtctaagatgacttgg**GC**tccgctgctactgtcttcc
Dchs1F79A Forward Primer	cggcagctcctctcatgtac**GC**catctctgcccaagagggcag
Dchs1F79A Reverse Primer	ctgccctcttgggcagagatg**GC**gtacatgagaggagctgccg
Dchs1R297A Forward Primer	tgacttacgagatcaaccgg**GC**gcagagcgagggtgatggacc
Dchs1R297A Reverse Primer	ggtccatcaccctcgctctgc**GC**ccggttgatctcgtaagtca

Supplementary Table 7: Oligonucleotides used to clone point mutations.
