# Supplementary material for: Structure of the planar cell polarity cadherins Fat4 and Dachsous1
Source: Nat Commun. 2023 Feb 16;14:891. doi: 10.1038/s41467-023-36435-x (PMC9935876; doi:10.1038/s41467-023-36435-x)
Supplement: Supplementary file 3 — Reporting Summary [file 41467_2023_36435_MOESM3_ESM.pdf]

nature portfolio

Corresponding author(s): Vincent C. Luca

Last updated by author(s): Dec 22, 2022

Reporting Summary

Nature Portfolio wishes to improve the reproducibility of the work that we publish. This form provides structure for consistency and transparency in reporting. For further information on Nature Portfolio policies, see our Editorial Policies and the Editorial Policy Checklist.

Statistics

For all statistical analyses, confirm that the following items are present in the figure legend, table legend, main text, or Methods section.

n/a	Confirmed	
☐	☒	The exact sample size (*n*) for each experimental group/condition, given as a discrete number and unit of measurement
☐	☒	A statement on whether measurements were taken from distinct samples or whether the same sample was measured repeatedly
☐	☒	The statistical test(s) used AND whether they are one- or two-sided<br>*Only common tests should be described solely by name; describe more complex techniques in the Methods section.*
☒	☐	A description of all covariates tested
☐	☒	A description of any assumptions or corrections, such as tests of normality and adjustment for multiple comparisons
☐	☒	A full description of the statistical parameters including central tendency (e.g. means) or other basic estimates (e.g. regression coefficient) AND variation (e.g. standard deviation) or associated estimates of uncertainty (e.g. confidence intervals)
☐	☒	For null hypothesis testing, the test statistic (e.g. *F*, *t*, *r*) with confidence intervals, effect sizes, degrees of freedom and *P* value noted<br>*Give P values as exact values whenever suitable.*
☒	☐	For Bayesian analysis, information on the choice of priors and Markov chain Monte Carlo settings
☒	☐	For hierarchical and complex designs, identification of the appropriate level for tests and full reporting of outcomes
☒	☐	Estimates of effect sizes (e.g. Cohen's *d*, Pearson's *r*), indicating how they were calculated

Our web collection on statistics for biologists contains articles on many of the points above.

Software and code

Policy information about availability of computer code

Data collection	X-ray data collection: NOMACHINE™. Chromatography: Unicorn 7.1 (GE Healthcare). MicroCal ITC200 (Malvern). Odyssey Fc (Licor). GelDoc XR + (Bio-Rad). BZ-X710 Confocal Microscope (Keyence). LSM880 Confocal Microscope (Leica Microsystems). Cytoflex5L flow cytometer (Beckman Coulter).
Data analysis	Data processing and structure refinement: XDS (Version March 15, 2019 BUILT=20190315), XDS (Version January 31, 2020, BUILT=20200131). Coot v0.8.9.1 EL, PyMOL v.1.7.2.1, phenix v1.17.1-3660.<br>ITC experiments: Origin7 v7.0552.<br>Structural analysis: Dali server, ccp4, PDBePISA server, Jalview v2.11.1.0., ConSurf server.<br>Confocal microscopy: Graphpad Prism v.7.0d, Keyence software v.1.3.0.3, ImageJ v1.53a, Leica software Zen 2.3 SP1 FP3 (black) v.14.0.22.201, Kaluza software v.2.1.

For manuscripts utilizing custom algorithms or software that are central to the research but not yet described in published literature, software must be made available to editors and reviewers. We strongly encourage code deposition in a community repository (e.g. GitHub). See the Nature Portfolio guidelines for submitting code & software for further information.

Data

Policy information about availability of data

All manuscripts must include a data availability statement. This statement should provide the following information, where applicable:

- Accession codes, unique identifiers, or web links for publicly available datasets
- A description of any restrictions on data availability
- For clinical datasets or third party data, please ensure that the statement adheres to our policy

The crystallography data generated in this study have been deposited in the Protein Data Bank under the accession code 8EGW [https://doi.org/10.2210/pdb8EGW/pdb] (Fat4(EC1-4):Dchs1(EC1-3)) and 8EGX [https://doi.org/10.2210/pdb8EGX/pdb] (Fat4(EC1-4):Dchs1(EC1-4)). Source data are provided with this paper. The MATLAB code used to analyze the co-culture experiments can be found at [https://github.com/dsprinzak/Medina-et-al].

Human research participants

Policy information about studies involving human research participants and Sex and Gender in Research.

Reporting on sex and gender	Not applied
Population characteristics	Not applied
Recruitment	Not applied
Ethics oversight	Not applied

Note that full information on the approval of the study protocol must also be provided in the manuscript.

Field-specific reporting

Please select the one below that is the best fit for your research. If you are not sure, read the appropriate sections before making your selection.

☒ Life sciences ☐ Behavioural & social sciences ☐ Ecological, evolutionary & environmental sciences

For a reference copy of the document with all sections, see nature.com/documents/nr-reporting-summary-flat.pdf

Life sciences study design

All studies must disclose on these points even when the disclosure is negative.

Sample size	No statistical method was used to determine sample size as it is not necessary for studies present in the manuscript. Samples used were sufficient for all parameters determined in this study. Cell staining, co-culture, and flow cytometry experiments were performed with three replicates with for at least two independent experiments. Crystallography data were collected for multiple crystals and the crystal with the best diffraction was used for structure determination.
Data exclusions	No data were excluded from analysis.
Replication	Each staining experiment was performed twice. Flow cytometry was performed twice using different cell passages. For phosphorylation studies, three different batches of protein were modified using the same conditions and were visualized using fresh stains. In vitro phosphorylation and Western blotting was performed twice. All experiments successfully reproduced initial results.
Randomization	For structure refinement, a subset of test reflections (5%) was selected at random for cross-validation. In the assays, cells were prepared from different batch of culturing for repeating experiments. For analysis, random field was chosen for the microscopy imaging.
Blinding	In all assays, cells were cultured and prepared for the experiment by the same person, thereby making blinding difficult. ITC binding experiments had control injections where the ligand was injected into buffer alone. For staining experiments, analysis of staining was blinded using control staining.

Reporting for specific materials, systems and methods

We require information from authors about some types of materials, experimental systems and methods used in many studies. Here, indicate whether each material, system or method listed is relevant to your study. If you are not sure if a list item applies to your research, read the appropriate section before selecting a response.

Materials & experimental systems

n/a	Involved in the study
☐	☒ Antibodies
☐	☒ Eukaryotic cell lines
☒	☐ Palaeontology and archaeology
☒	☐ Animals and other organisms
☒	☐ Clinical data
☒	☐ Dual use research of concern

Methods

n/a	Involved in the study
☒	☐ ChIP-seq
☐	☒ Flow cytometry
☒	☐ MRI-based neuroimaging

Antibodies

Antibodies used	Goat anti-6-His HRP Conjugate (A190-113P, Lot# 22) (Bethyl Laboratories)
Validation	Validation by the company was done by recombinant production of a 6-His tagged protein in E. coli, followed by blotting with A190-113P. Validation report can be downloaded from the manufacturer's website.

Eukaryotic cell lines

Policy information about cell lines and Sex and Gender in Research

Cell line source(s)	HEK293 cells transduced with Fat4-mCitrine fusion protein. HEK293 cells transduced with Dchs1-mCherry fusion protein. Both provided by David Sprinzak lab. Trichoplusia ni cells were purchased from Expression Systems and were cultured using ESF921 media from Expression Systems.
Authentication	Authentication performed by Sprinzak lab (see Olga et al. eLife, 2017.) Authentication of Trichoplusia ni cells can be found on the Expression Systems website.
Mycoplasma contamination	All cell lines tested negative for mycoplasma contamination.
Commonly misidentified lines (See ICLAC register)	HEK293 cells are commonly misidentified according to ICLAC. The cell line in particular was not important as HEK293 cells do not endogenously express Fat4 or Dchs1 (see Olga, et al. eLife, 2017.).

Flow Cytometry

Plots

Confirm that:

☒ The axis labels state the marker and fluorochrome used (e.g. CD4-FITC).

☒ The axis scales are clearly visible. Include numbers along axes only for bottom left plot of group (a 'group' is an analysis of identical markers).

☒ All plots are contour plots with outliers or pseudocolor plots.

☒ A numerical value for number of cells or percentage (with statistics) is provided.

Methodology

Sample preparation	HEK293 cells were seeded in 24-well plates at 70% confluence for 24 hours. Directly prior to FACS, cells were trypsinized, spun at 1000 rpm for 5 minutes, and resuspended in 200 uL of PBS with 1% FBS and 5 mM EDTA.
Instrument	Beckman Coulter Cytoflex5L flow cytometer
Software	Beckman Coulter Kaluza flow cytometry analysis software.
Cell population abundance	No sorting was performed. Relevant cell populations constituted all of the cells evaluated.
Gating strategy	Only identification of the cell population was necessary. Fluorophore expression is endogenous to the cell lines evaluated and only one fluorophore was examined.

☒ Tick this box to confirm that a figure exemplifying the gating strategy is provided in the Supplementary Information.
